# Omani senior secondary school students’ knowledge of and attitudes to antibiotic resistance

**DOI:** 10.1371/journal.pone.0264500

**Published:** 2022-02-25

**Authors:** Abdullah Ambusaidi, Neil Taylor, Frances Quinn, Nadya Rizk, Subhashni Taylor

**Affiliations:** 1 Sultan Qaboos University, Muscat, Sultanate of Oman; 2 University of New England, Armidale, NSW, Australia; 3 College of Arts, Society & Education, James Cook University, Smithfield, Cairns, QLD, Australia; University of Connecticut, UNITED STATES

## Abstract

Antibiotic resistance is a worldwide problem that is increasing largely due to the misuse of antibiotics in human health and agriculture. This situation is further exacerbated by a dearth of new antibiotic development, the focus of pharmaceutical companies having shifted to more lucrative treatments for chronic conditions such as elevated blood pressure. To conserve the efficacy of the current crop of antibiotics, it is vital that they are used appropriately by individuals. Effective education may be a means to achieve such appropriate use. This paper reports on a large-scale, mixed methods study, which employed a survey and oral questionnaires, undertaken with senior secondary Omani students. The study explored students’ understanding of antibiotic resistance as well as their attitudes to the issue of antibiotic resistance. The study findings indicated that, although some students had a reasonably clear understanding of antibiotic resistance, many had serious misconceptions that could result in misuse of antibiotics. The article concludes with suggestions for amending secondary school pedagogy in Oman to address the misconceptions.

## Introduction

In August 2019, the chief medical adviser to the UK Government stated that antibiotic resistance endangers at least 10 million people every year and could kill us before climate change does [1]. Such resistance is caused by the misuse of antibiotics in humans and other animals and could render the drugs ineffective. Several studies, most carried out in the Western context, have examined the causes of misuse of antibiotics, often finding some association with lack of knowledge [2–4].

This article reports on a study that examined the understanding and attitudes towards antibiotic resistance amongst a cohort (n = 952) of senior secondary students in the Sultanate of Oman. It begins by providing an overview of antibiotic misuse and public understanding of antibiotic resistance before describing the methodology and findings from the Oman study. The article concludes with a discussion of the findings, particularly the misconceptions concerning antibiotics among students and how changes to the school curriculum might address such misconceptions.

## Literature review

Less than a century ago, individuals were dying of bacterial infections and diseases that are treatable today. For example, pneumonia and tuberculosis killed approximately 300,000 Americans in 1930 and accounted for 22% of total deaths, but deaths from severe pneumonia infections declined by 60–75% when antibiotics were introduced in the late 1930s [5]. Unfortunately, the usefulness of antibiotics in the treatment of infections is being increasingly compromised by their misuse (particularly by their overuse), resulting in antibiotic resistance and the appearance of highly resistant bacteria (so-called superbugs) against which the range of conventional antibiotics is ineffective [6,7]. Antibiotic resistance represents one of the biggest threats to global health today [8], with antibiotic-resistant infections affecting more than 2 million people each year and accounting for at least 23,000 deaths, with a consequent huge economic burden [9].

Several studies have been conducted to clarify the extent of and reasons for the misuse of antibiotics. For example, in 2015, the World Health Organisation (WHO) conducted a large-scale, cross national 14-question survey about individuals’ knowledge about antibiotic resistance [8]. The study represented the views of a total of 9,772 participants aged 16–65 years from 12 countries, selected to represent a diversity of level of development, population, and other characteristics (Nigeria, South Africa, Barbados, Mexico, India, Indonesia, Egypt, Sudan, Russia, Serbia, China and Vietnam). The study found that one contributing factor to antibiotic misuse is the widespread misconception that antibiotics are effective for the treatment of viral conditions, such as the common cold (reported by 64% of respondents). Other studies involving the US public [10,11], student health care professionals in Italy [12], university students in Italy [13] and the general public in Poland [14] have also reported significant levels of this misconception. Responses from 25% of participants in the WHO study indicated that inappropriate access to antibiotics through relatives or without a prescription also contributed to incorrect use and consequent resistance. Willingness to take antibiotics without a prescription has also been documented in other studies, for example, among young Italians [2]. The WHO survey found that another contributing factor to antibiotic misuse is failure to take the full course of antibiotics, reported by 32% of respondents.

The findings from the WHO and other studies beg the question of the extent to which members of the public understand the link between antibiotic misuse and the development of antibiotic-resistant bacteria. The findings of the WHO survey indicated that, in general, the participants were aware of the problem of antibiotic resistance but did not fully understand what caused it or what to do about it. A small study of public understanding of this issue in the US indicated that the link between misuse and resistance was recognized by most participants but only 30% felt that antibiotic resistance was a significant problem or that their personal actions might influence it [15]. Furthermore, over one-third believed that people rather than bacteria become resistant to the antibiotics.

Although the WHO study did not include countries from the Middle East and Gulf Region, other studies indicate that misconceptions about and misuse of antibiotics is also prevalent in these regions. For example, in a study conducted in the north of Jordan where just over 40% of 1,060 randomly selected adults had taken oral antibiotics in the previous two months, 38% acquired the antibiotic without a prescription and 32% did not complete the recommended course of treatment [16]. In addition, 70% of the sample did not know the meaning of the term antimicrobial resistance and between 39% and 50% of the sample held misconceptions about antibiotics and/or revealed misuse of antibiotics. Similar findings have been reported in studies conducted with participants from Kuwait [17], Saudi Arabia [18–21], Yemen [22,23], and Palestine [24].

A review of nine studies (from January 2000 to June 2017) undertaken in Gulf Cooperation Council countries found that there were many misconceptions concerning the efficacy of antibiotics, with nearly 40% of participants incorrectly believing that they were effective in treating minor ailments such as the common cold and coughs [25], and 44% of participants were unaware that antibiotics have no significant effect on viruses. The authors believed that this lack of knowledge frequently resulted in the over prescription of antibiotics because patients often influence whether physicians will prescribe antibiotics. The review also revealed that a median of 37% of study participants obtained antibiotics from family members or friends, indicating they had received them without a medical consultation. Furthermore, nearly half the respondents stored left-over antibiotics at home for future use–often beyond their expiry date. A similar finding was reported in a study conducted in Qatar by Alkhuzaei et al. [26] which assessed the knowledge, attitude, and practice of antibiotic use among 722 adult patients attending primary health care centres. Over 87% of these patients indicated they pressured physicians to prescribe antibiotics.

### Study rationale and aims

The first author of this study is an Omani citizen, and all authors have an interest in and commitment to public understanding of science and education. Very few studies have been conducted on antibiotics use among the public in Oman. Two hospital-based studies conducted in Oman found that prescribing guidelines aimed at diminishing antibiotic resistance were not always followed [25,27]. The only study of the general Omani public of which we are aware is a small-scale interview-based study of 21 people which revealed that individuals were erroneously taking antibiotics for the common cold, and only some participants were aware of the administration instructions for antibiotics (such as dosage and duration) while others lacked an understanding of the risk of non-adherence. Moreover, people who were demanding antibiotics showed a preference for visiting a private doctor for easy and fast access to antibiotics even if they had to pay for the cost of the visit and the prescription [28].

The three studies indicate that Oman likely shares similar issues in relation to antibiotic resistance as do other countries. The study reported in this paper aims to increase understanding of use of antibiotics in Oman with the objective of providing a basis for the introduction of health and education policy initiatives to improve public knowledge about antibiotics use. In addition, this study includes senior secondary students, thus extending an understanding of public knowledge and behaviours related to antibiotic use in the Gulf Countries [29] from the predominantly adult participant base of previous studies.

In particular, this study aims to extend a version of the WHO survey to the Omani context and address the concern expressed in the review into antibiotic use in the Gulf Countries: “a multi-disciplinary approach must be put in place to educate the public on appropriate antibiotic use” [25]. The cohort of secondary school students was chosen since it may be possible to change the school curriculum to better educate and engage students in this age in relation to antibiotic use and, by extension, influence their choices and the choices of family members.

### Research context

The Sultanate of Oman, in the south-east of the Arabian Peninsula, has a middle-income economy that is heavily dependent on dwindling oil reserves. The country’s political leadership has actively pursued a development plan that focuses on diversification, industrialization, and privatization, with the objective of generating more job opportunities and reducing the oil sector’s contribution to Oman’s Gross Domestic Product [30]. Animal husbandry is limited in Oman and most animal products, mainly meat and milk, are imported, so antibiotic use in the agricultural sector contributes little to possible resistance development. There is an aquaculture sector, and the Oman Ministry of Agriculture and Fisheries has been proactive in developing probiotic and herbal treatments for the sector to limit resistance issues associated with antibiotic use in that industry [31]. There is ready access in Oman to broad-spectrum antibiotics for humans [27].

Oman’s population of approximately 4.6 million people is growing at approximately 3.1% per annum, with about half of the population being under 30 years of age. Education is free and compulsory until grade 10 (age 16) and remains free but non-compulsory until grade 12 (age 18). Secondary schools in Oman are segregated by gender.

The school curriculum addresses the topic of antibiotics in grade 6, when students learn about Alexander Fleming and his discovery of penicillin, but misuse of and resistance to antibiotics is not covered. In the grade 11 Science and Environment Science Curriculum, there is some coverage of antibiotics, including issues of misuse and resistance [32]. However, due to different subject specializations, non-science students would not receive this information.

## Methodology

This research was conducted with Omani Secondary level students aged 15–17 years old. It employed a mixed-methods methodology involving a quantitative survey (n = 952) followed by oral questionnaires (n = 107). The research was reviewed and approved by The Ministry of Education Technical Office for Studies and Development (project number 281978993), Department of School Counselling at the Ministry of Education (MoE) and the Department of School Health at the Ministry of Health (MoH). Consent, as consistent with Oman MoE approval, was implied, as no personal details were collected, the research is very low risk and the burden of participation minimal. Participants were informed about the nature and purpose of the research prior to their involvement.

The survey and oral questionnaires explored the following research questions:

- What are students’ behaviours in relation to taking antibiotics? (Survey questions 1–5, Oral questionnaire question 2)
- What are students’ understandings of antibiotics and antibiotic resistance and the factors that contribute to antibiotic resistance? (Survey questions 6–13 and 16, Oral questionnaire questions 1 and 3)
- What are students’ attitudes towards antibiotic use? (Survey questions 14, 15 and 17, Oral questionnaire question 4)

### Survey

The survey (see S1 Appendix A. Survey) was an adaptation of the WHO instrument [8], designed to capture descriptive frequency data. The adaptations included the addition of demographic questions related to gender, age and rurality of the school attended, together with questions 4 and 17 and an open-ended question on the importance of learning about the use of antibiotics in schools. In addition, the phrase “school curriculum” was included as an option for questions 9 to 12. The instrument was translated into Arabic by the first author (AA) then checked by an English and Arabic Language specialist. The specialist checked the Arabic version and back translated it into English. The back translated version was also checked by one of the authors (NR) to ensure validity of the translation. The Arabic version was further trialled using a number of senior secondary classes in Omani schools as well as specialists in the schools of health education in both the Ministries of Education and Health to check the content and translation validity. A similar approach to adapting this WHO survey was employed by Prigitano et al. in a study with Italian university students [13].

### Oral questionnaire

At the completion of the survey, an oral questionnaire was conducted with a randomly selected student subset of the survey sample (107 students).

The oral questionnaire comprised 5 questions aligned to the research questions (see S2 Appendix B. Oral Questionnaire Protocol) to enable a more in-depth understanding of students’ knowledge of antibiotics and antibiotic resistance. The oral questionnaires were administered by research assistants proficient in Arabic and English. The research assistants were not involved in teaching the participants, to negate conflict of interest issues.

### Participants

The survey was administered to a cohort of 952 students comprising male and female students in 20 secondary schools (grades 10–11) in urban and rural areas of all educational regions. The sample comprised 10 all-male and 10 all-female schools. The schools were selected to represent each of the eleven educational governors (directorates) in the Sultanate of Oman that offered grades 10–11. The school principals invited students in grades 10–11 to take part in the survey. A total of 1000 students were enrolled in the selected 20 schools and 952 students completed a paper-based survey giving a very high response rate of approximately 95%. Oral questionnaires were conducted with 107 students. The questionnaires were conducted in Arabic then translated into English by the research assistants. The translation was checked by two authors (AA and NR). Table 1 summarizes the demographic data for the survey and questionnaire samples.

**Table 1:** Demographics of participants.

Instrument		Urbanization		Gender		Age (Years)			
		Urban	Rural	Male	Female	15	16	17	>17
Survey	Number	594	358	385	567	219	341	303	89
	Percentage	62.4	37.6	40.4	59.6	23	35.8	31.8	9.3
Oral Questionnaire	Number	47	60	52	55	Students: 15–16 years			
	Percentage	43.9	56.1	48.6	51.4				

The relevant part of the official Grade 11 Oman textbook was also translated into English and all authors read through the material to understand the content concerning antibiotics and the way in which it was presented.

### Data analysis

Statistical analysis of data was undertaken using the IBM SPSS Statistics for Windows Version 25 software package. Descriptive statistics were largely employed in the analysis to present certain findings graphically and, where appropriate, chi square tests were performed to compare frequencies between genders and rural/urban populations. The strength of association was also calculated using Cramer’s V test. For all tests, p-values of 0.05 or less were considered to be statistically significant. Interpretation criteria for Cramers’ V statistics are outlined in Table 2 and significant differences are only reported for small effect sizes.

**Table 2:** Interpretation criteria for Cramer’s V measure of effect size for chi-square contingency tables [33].

Degrees of freedom	Cramer’s V statistic	Effect size
**1**	0.10<V<0.30	Small
	0.30<V<0.50	Medium
	V>0.50	Large
**2**	0.07<V<0.21	Small
	0.21<V<0.35	Medium
	V>0.35	Large
**3**	0.06<V<0.17	Small
	0.17<V<0.29	Medium
	V>0.29	Large

Data from the oral questionnaire was content analysed to capture and qualify the conceptions that students expressed in relation to (1) defining antibiotics and antibiotic resistance, (2) using antibiotics, and (3) attitudes towards antibiotics use. The researchers were particularly interested in identifying the most prevalent conceptions and misconceptions that students expressed about each of these three categories.

Two researchers (NR and FQ) developed a scheme for coding the responses to the oral questionnaire. *A priori* codes were used, and for each question, a specific set of codes was adopted. To establish inter-coder reliability, for each question, the two researchers coded 20 responses separately. They then cross-checked their coding, negotiated and refined and added to their codes as necessary. Afterwards, the two researchers independently coded 15 additional entries and used those results to calculate an inter-coder reliability score, which provided higher than 90% on every occasion. Once all questions were coded in this way, one of the researchers (NR) completed the coding of the remainder of the dataset.

## Findings

Responses to survey questions are reported in this section with specific reference to each question of the survey (see S1 Appendix A. Survey). Data revealed a number of significant misconceptions concerning antibiotics as well as differences in understanding of how antibiotics work and how they should be used. Some significant differences in these factors were evident between males and females, and students from urban and rural areas, which, though only small, recurred across several survey items. Data from the oral questionnaire provided triangulation of the quantitative findings and further insights.

### Behaviour in relation to antibiotics use

A total of 386 (41%) participants claimed to have taken antibiotics during the previous month before the survey (Survey Q1). This rose to 56% for the previous six months. Most students (65%) had obtained their antibiotics via a medical script obtained from a doctor (Survey Q2), while 17% of the cohort obtained antibiotics by other means (Survey Q2). Sixty percent of respondents received advice from health care providers on how to take their antibiotics in terms of dosage and duration (Survey Q3). This is broadly consistent with the proportion being prescribed antibiotics by their doctor, but only half (50%) of students claimed that their parents reinforced the prescription instructions (Survey Q4). Sixty-nine percent of students claimed that antibiotics were purchased from a pharmacy and only 6% mentioned that they took antibiotics left over from a previous prescription (Survey Q5).

Some significant but small differences between urban and rural participants were evident relating to these results. A total of 63.1% of urban compared to 55% of rural participants indicated that they had received advice on dosage and timing by a doctor (*X*^*2*^(2) = 6.244, *p* = .044, *V* = .081). Similarly, 52.9% of urban compared to 44.4% of rural participants indicated that they had received advice on dosage and timing from their parents (*X*^*2*^(2) = 6.506, *p* = .039, *V* = .083). This is suggestive of less available professional advice in rural areas, but the difference is only small.

These survey findings were generally consistent with responses to question 2 in the oral questionnaire: ‘Would you take antibiotics without a prescription? Why/why not? (Table 3).

**Table 3:** Findings pertaining to Question 2: Would you take antibiotics without a prescription? Why/Why not?.

	Yes		No	
	Reason	Count	Reason	Count
Valid			Because antibiotics can have negative side effects on my body	17
			Because a doctor has to identify the antibiotic that is useful in each specific situation	10
			Because I wouldn’t know the dosage	5
			Because this may lead to health complications	4
			Because they can be ineffective	2
			Because this can be dangerous	2
			Because they have a use-by date	1
			So I can ensure they don’t affect my body negatively	1
			Because I would need to follow the correct instructions	1
Invalid	Because they relieve the pain in time of need	7	Because excessive use of antibiotics can compromise the body’s immune system	1
	Because if I already used them, then I already know what they are	1	Because this would make me more susceptible to other diseases	1
	Because it would be an emergency situation	1		
Unclear	Because I do not go to the doctor	1	Because antibiotics are/can be harmful	15
			So the diseases do not evolve or multiply	4
Missing		13		19
Total		23*		83

*One respondent indicated “yes, but I know it’s the wrong thing to do”; not included in table.

Seventy-eight percent of students reported that they would not take antibiotics without a prescription while 22% responded with sometimes or yes. The main reason given for taking antibiotics without a prescription was: “Yes, to reduce the pain”. Interestingly, of those who answered “no”, around half (47%) offered an unclear or no reason to justify their decision, while the other half (53%) presented valid supportive reasons.

### Understanding of antibiotics

These results relate to questions 6 to 8 and 16 of the survey, which explore participants’ understanding about the use of antibiotics and questions 1 and 3 of the oral questionnaire. Although 60% of students had received advice from health professions on dosage and duration (Survey Q3), only 38% of students correctly responded that they should complete the full course of antibiotics, while 48% believed they could stop the medication when they felt better and 14% did not know (Survey Q6). Sixty four percent of respondents identified as medically incorrect the following statement: “It is OK to use antibiotics that were prescribed to someone else as long as the disease that is being treated for is the same”, with only 20% believing this practice was appropriate (Survey Q7)).

In relation to these questions, some small differences were apparent between genders. A small significant finding was that slightly fewer males (45.5%) than females (50.1%) indicated incorrectly that they should stop taking antibiotics when they felt better (*X*^*2*^ (2) = 10.870, *p* = .004, *V* = .107). On the other hand, significantly fewer males (51.9%) than females (71.6%) identified as incorrect the statement: “It is OK to use antibiotics that were prescribed to someone else as long as the disease that is being treated for is the same”. This was a medium strength association (*X*^*2*^ (2) = 44.901, *p* < .001, *V* = .217).

A surprising misconception emerged when the response to the first question in the oral questionnaire was scrutinised: “Can you tell me what you know about antibiotics, what they are and how do they work?” Many students thought that antibiotics were analgesics (See Table 5).

**Fig 1:**
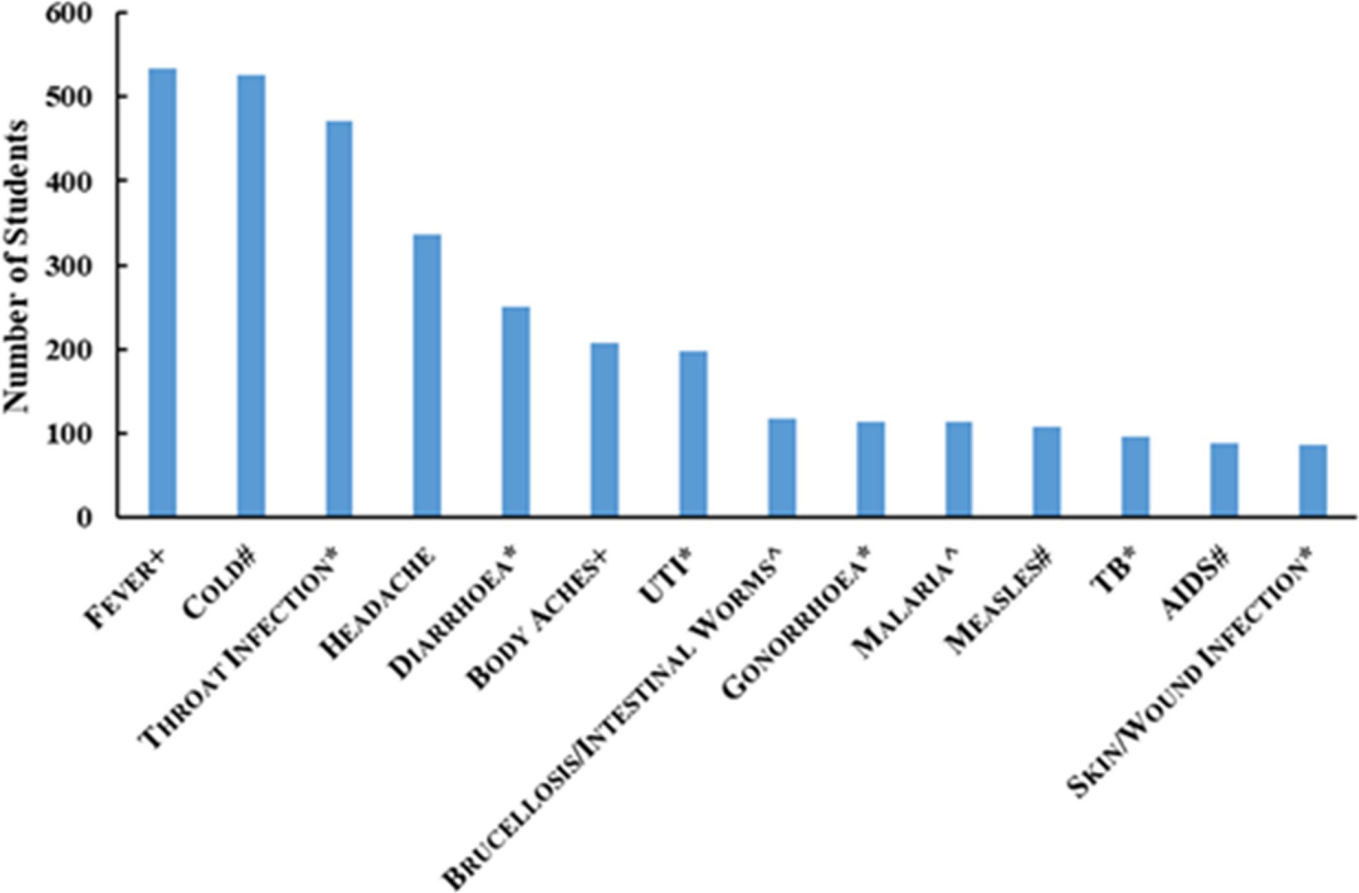
Participants’ identification of conditions they thought were treatable with antibiotics (^+^could be either viral or bacterial; ^#^viral; *bacterial; ^^^parasitic).

Participants’ identification of conditions they thought were treatable with antibiotics (Survey Q8, summarised in Fig 1), provides a somewhat alarming picture of the level of knowledge of the medical utility of antibiotics. Fever, colds, headaches and throat infections were the four conditions most frequently endorsed by the students as being treatable with antibiotics. Conversely, TB (a bacterial disease) and skin/wound infections (most commonly bacterial) were the least frequently identified as treatable with antibiotics.

In relation to knowledge about whether particular conditions are treatable or not with antibiotics, some small significant differences between urban/rural and male/female participants and male and female participants are summarized in Table 4.

**Table 4:** Percentages of urban/rural, and male/female students identifying whether conditions are treatable or not with antibiotics.

Condition	Correctness of response	Urban	Rural	*X*^*2*^_(1)_ Statistic	Male	Female	*X*^*2*^_(1)_ Statistic
AIDs not treatable	correct	93.4	86	14.437, *p* < .001, *V* = .123	nsd	Nsd	-
Common cold treatable	incorrect	59.3	48.3	10.799, *p* = .001, *V* = .107	49.6	58.9	8.011, *p* = .005, *V* = .092
Measles: not treatable	correct	91.4	84.1	11.952, *p* = .001, *V* = .112	nsd	Nsd	-
Throat infections	Depends on causal factor	47.8	55.3	5.021, *p* = .025, *V* = .073	58.2	45.5	19.393, *p* < .001, *V* = .143
TB not treatable	incorrect	92.9	84.9	15.818, *p* < .001, *V* = .129	85.5	92.9	14.190, *p* < .001, *V* = .122
Gonorrhea not treatable	incorrect	Nsd	Nsd	-	83.1	91.4	14.774, *p* < .001, *V* = .125
Fever treatable	(generally incorrect, some exceptions)	Nsd	Nsd	-	51.4	36	19.393, *p* < .001, *V* = .143

As indicated in Table 4, there are no overall or recurring patterns of difference in the correctness or otherwise of their understanding in either the demographic variables tested.

These survey findings were generally consistent with responses to the first question of the oral questionnaire: “Can you tell me what you know about antibiotics–what they are and how they work?” which are summarised in Table 5. Sixteen percent of students reported that antibiotics were analgesics and treated symptoms such as pain and fever. Thirty-three percent of students correctly responded that antibiotics acted against bacteria; for example, “a medicine that works on eliminating bacteria” or “a medicine that attack[s] the bacteria in the body”, however 21% believed that antibiotics acted against viruses. Some students had more elaborate theories about antibiotics such as” “Chemical compounds extracted from bacteria and work to eliminate pathogens; synthetic lymphocytes that attack the virus or bacteria”.

**Table 5:** Findings pertaining to oral questionnaire Question 1: Can you tell me what you know about antibiotics—what they are and how they work?.

		Statement	Count*
Misconceptions	Function or role of antibiotics	Antibiotics are analgesics	17
		Antibiotics are anti-viral; they kill viruses	11
		Antibiotics stimulate and/or boost the body’s immunity	11
		Antibiotics kill infections	2
		Antibiotics stimulate the non-infected cells to fight the infected cells	1
		Antibiotics are used to reduce the temperature of the body	1
		Antibiotics cure injuries	1
	Description or nature of antibiotics	Antibiotics are chemicals	6
		Antibiotics are antibodies	2
		Antibiotics are synthetic lymphatic cells	1
		Antibiotics are useful bacteria	1
		Antibiotics are dead viruses	1
Valid conceptions	Function or role of antibiotics	Antibiotics kill/fight bacteria	37
		Antibiotics help reduce illness/ are a treatment used to fight disease	17
		Antibiotics kill/destroy the causes of disease	4
	Description or nature of antibiotics	Antibiotics are medications used in emergencies and/or special circumstances	8
		Antibiotics are living creatures	7
	Behaviour or antibiotic use	Antibiotics are medicines that you can ingest (in pill form), drink (in liquid form), or receive via injection.	7
		Antibiotics are administered according to a medical prescription	4

*Some responses featured more than one statement and were assigned more than one code.

The breadth of misconceptions that students expressed when describing antibiotics is also apparent in Table 5. These misconceptions mostly relate to the function and nature of antibiotics.

In relation to use of antibiotics in agriculture (Survey Q16), almost equal numbers of respondents, (just over 40%,) claimed “yes” or “I don’t know”, and about 20% responded “no” to the question of whether antibiotics are intensively used in the agricultural sector in Oman. None of the responses to the oral questionnaire featured concepts relevant to the use of antibiotics in agriculture.

### Awareness of key terms

These results pertain to Questions 9–12 of the survey (see S1 Appendix A. Survey), which focus on four key terms related to antibiotic resistance. Student awareness of key terms is indicated in Table 6, which shows that just over half of the students had come across the term antibiotic resistance, and fewer were aware of superbugs, drug resistance and antimicrobial resistance.

**Table 6:** Participant awareness of terms associated with antibiotic resistance.

Item #	Term	% having heard of the term
9a	Antibiotic resistance	52
10a	Superbugs	35
11a	Drug resistance	43
12a	Antimicrobial resistance	36

Sources of information about these terms reported by students were ranked from most to least frequent, as listed in Table 7. Doctors were the most frequently cited source of information, with media and school curriculum second and third most frequent respectively.

**Table 7:** Sources of information associated with antibiotic (Survey Qs 9–11).

Source	Rank			
	Antibiotic resistance	Superbug (principal bacteria)	Drug resistance	Antimicrobial resistance
Doctor	1	1	1	1
Media (TV, radio, newspapers or other, in addition to social media)	4	2	2	2
School curriculum	2	3	4	3
Nurse	3	4	3	4
Friend or relative	5	6	5	5
Awareness campaign	6	5	6	6
Pharmacist	7	7	7	7
I don’t remember	8	8	8	8
Other	9	9	9	9

As indicated by Table 7, the school curriculum is a relatively important source of information for students about antibiotic resistance. No meaningful significant differences were detected across the demographic variables for the sources of information indicated by students.

### Understanding of antibiotic resistance

Student knowledge of antibiotic resistance tapped by Survey questions 13–15 is outlined in this section, commencing with students’ identification of a series of statements related to antibiotic resistance (Survey Q13) as either correct or incorrect. The proportion of students accurately indicating whether these statements were correct or not is shown in Table 8, ordered from most to least well understood.

**Table 8:** Percentage of participant with correct responses to statements about antibiotic resistance (Survey Q13).

	Statement	% students correct
h.	Infections caused by bacteria that are antibiotics resistant can make some medical procedures such as surgery, organs transplant, and cancer treatments much more dangerous than they were	68
e.	Antibiotics resistance is an issue in other countries but not in Oman	64
b.	A lot of types of infections have become increasingly resistant to antibiotics	64
d.	Antibiotics resistance is an issue that could affect me or my family	60
g.	Bacteria that are antibiotic resistant can be passed from one person to another	58
c.	If a bacterium is resistant to antibiotics, then the cure for the infection caused by the bacterium becomes extremely difficult or impossible	56
f.	Antibiotics resistance is an issue for those who consume antibiotics regularly	39
j.	It is possible to cure diseases related to viruses through using antibiotics	38
i.	Antibiotic resistance can be overtaken by increasing the dose of the medication	35
a.	Antibiotic resistance occurs when your body becomes resistant to the positive effects of antibiotics, and antibiotics no longer work as they should	27

As shown in Table 8, while most students could correctly respond to items about the prevalence and potential impact of antibiotic resistance (items h, e, b and d), there was a widespread erroneous belief that the human body rather than the bacterium becomes resistant to antibiotics (73% incorrect). This is consistent with findings from previous studies (e.g. [15]). Once again, a majority of students (62%) expressed the previously documented misconception found in the WHO study [8] that antibiotics were effective against viral infections, while only 35% of students correctly believed that an increased dose of antibiotics could be used to overcome resistance.

The Chi square tests of associations between urban/rural participants and gender and Omani students’ understanding of the concept of antibiotic resistance tapped by Survey Q13 detected three small significant associations. Significantly more urban (59.6%) than rural (48.9%) participants correctly identified the statement “if a bacterium is resistant to antibiotics, then the cure for the infection caused by the bacterium becomes extremely difficult or impossible” as correct (*X*^*2*^ (1) = 10.384, *p* = .001, *V* = .104). In relation to gender differences, fewer males (56.9%) identified as incorrect the statement “antibiotics resistance is an issue in other countries but not in Oman” than females (69.1%) (*X*^*2*^ (1) = 14.974, *p* < .001, *V* = .125). Similarly, fewer males (55.6%) than females (65.8%) identified the statement: “It is possible to cure diseases related to viruses through using antibiotics” as incorrect (*X*^*2*^ (1) = 10.092, *p* = .001, *V* = .103). However, these gender differences are only small, and only evident for two of the 10 items.

### Attitudes to antibiotic use and resistance

**Fig 2:**
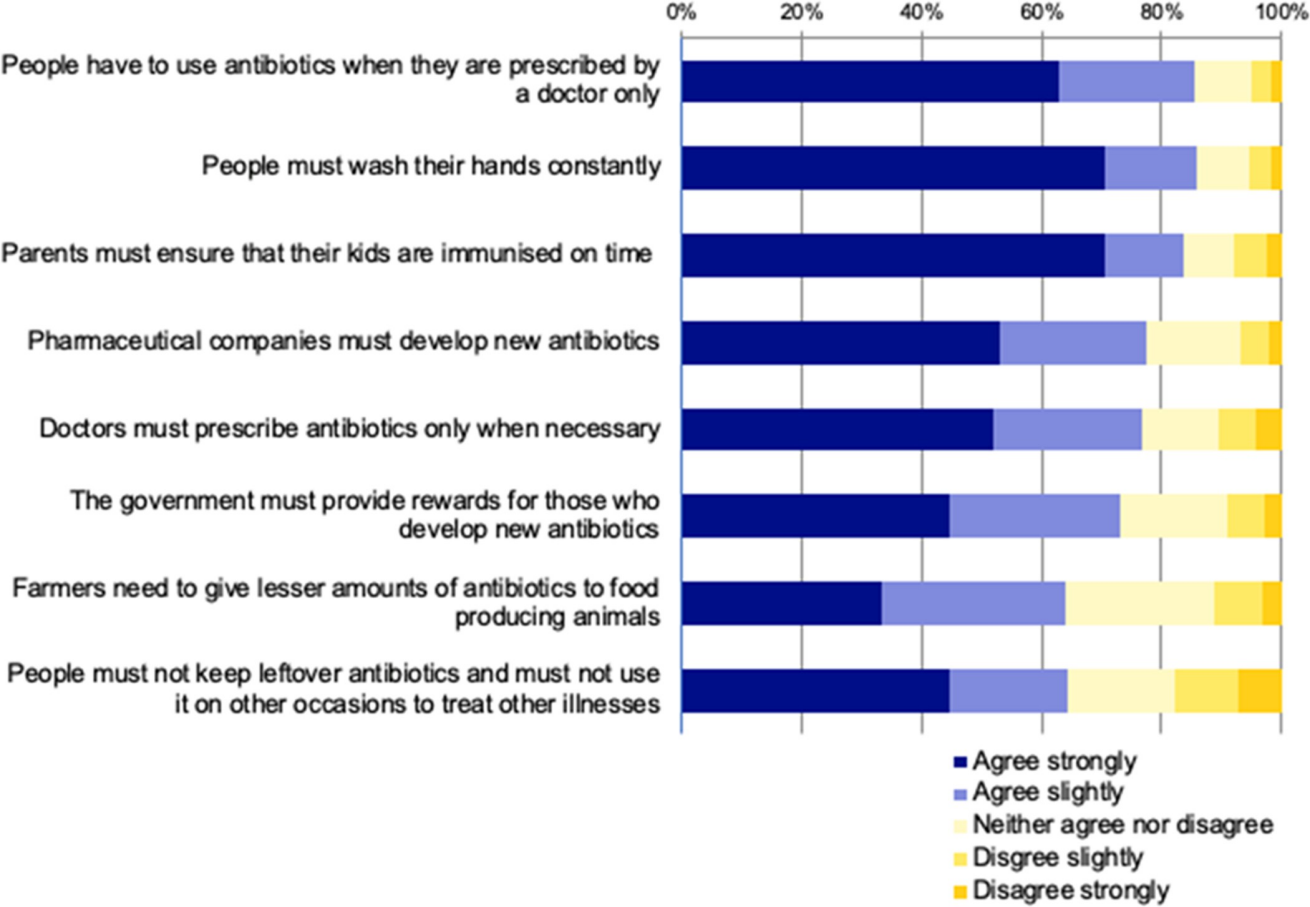
Participants’ attitudes towards how to address the issue of antibiotic resistance.

Students’ perceptions of ways to address antibiotic resistance (Survey Q14) are summarised in Fig 2, which shows that the majority of students agreed to some extent with all of the propositions.

The most strongly endorsed items were that people should only use antibiotics on prescription by a doctor, wash hands constantly and ensure that their children are immunised on time. Hence the students appear to be focussing on the responsibility of individual antibiotic users to combat antibiotic resistance. The important roles of other people and organisations, such as pharmaceutical companies, doctors, the government and farmers, was less frequently recognised. Finally, the least frequently endorsed item about not keeping and reusing leftover antibiotics indicates that this is an area of potential traction in policy and educational messaging related to antibiotic resistance.

The question of personal responsibility was further tapped by the second question of the oral questionnaire: “Would you take antibiotics without a prescription? Why/why not?” Only 15 of the 107 students claimed they would take antibiotics without a prescription, generally citing the need to do this in some kind of emergency, in some cases inaccurately again confusing antibiotics with analgesics; “Yes, because they inhibit pain in times of need”.

In response to the third question in the oral questionnaire “Do you understand what is meant by antibiotic resistance? How does this develop?” the respondents’ ideas were expressed in relation to what resistance is, what causes it and what it results in. These results are summarised in Table 9. While 17% of respondents expressed no knowledge of the concept of antibiotic resistance, around 40% of the statements expressed were accurate. Interestingly, 22% of questionnaire respondents incorrectly described resistance as developed by the human body rather than the pathogen. Indicative quotes include: “When the body resist[s] the antibiotic by using it several times” and “When frequent use the body will start resisting the antibiotic”. However, 27% displayed some understanding that antibiotic resistance developed in the pathogen rather than the host; for example: “When taking antibiotics significantly, the bacteria are resistant and are not affected by the antibiotic”, and “The continuous use of antibiotics will make the pathogenic bacteria recognise it and therefore resist it and will not be able to show the effect of the patient”. No one actually linked antibiotic resistance to failure to complete a full course of the drugs, although one student did suggest how resistance could be overcome: “When the bacteria overcome the antibiotics so a stronger antibiotic must be used”.

**Table 9:** Findings pertaining to Question 3: Do you understand what is meant by antibiotic resistance? How does this develop?.

		Statement	Count*
Misconceptions	Description	Antibiotic resistance is the resistance or immunity that the body acquires after getting the antibiotic	10
		Antibiotic resistance is the antibiotic’s resistance to and destruction of bacteria	7
		Antibiotic resistance is when the body adapts to the bacteria	1
		Antibiotic resistance is a person’s resistance to using antibiotics given its dangers	1
	Cause	Antibiotic resistance is when the virus resists the antibiotics	3
		Antibiotic resistance is when the bacterium creates antibodies against the antibiotic	2
	Result	Antibiotic resistance is when the body resists and no longer accepts/responds to the antibiotic	24
		Antibiotic resistance occurs when the pain disappears	2
Accurate conceptions	Description	Antibiotic resistance occurs when the bacteria resists the antibiotics	29
	Cause	Antibiotic resistance occurs due to the excessive use of the same antibiotics	14
	Result	Antibiotic resistance occurs when the antibiotics become ineffective	8
		Antibiotic resistance occurs when we need to use a stronger dosage of the antibiotics or a different antibiotic	2
I do not know			18

*Some responses featured more than one statement and were assigned more than one code (total of 131 statements were coded).

**Fig 3:**
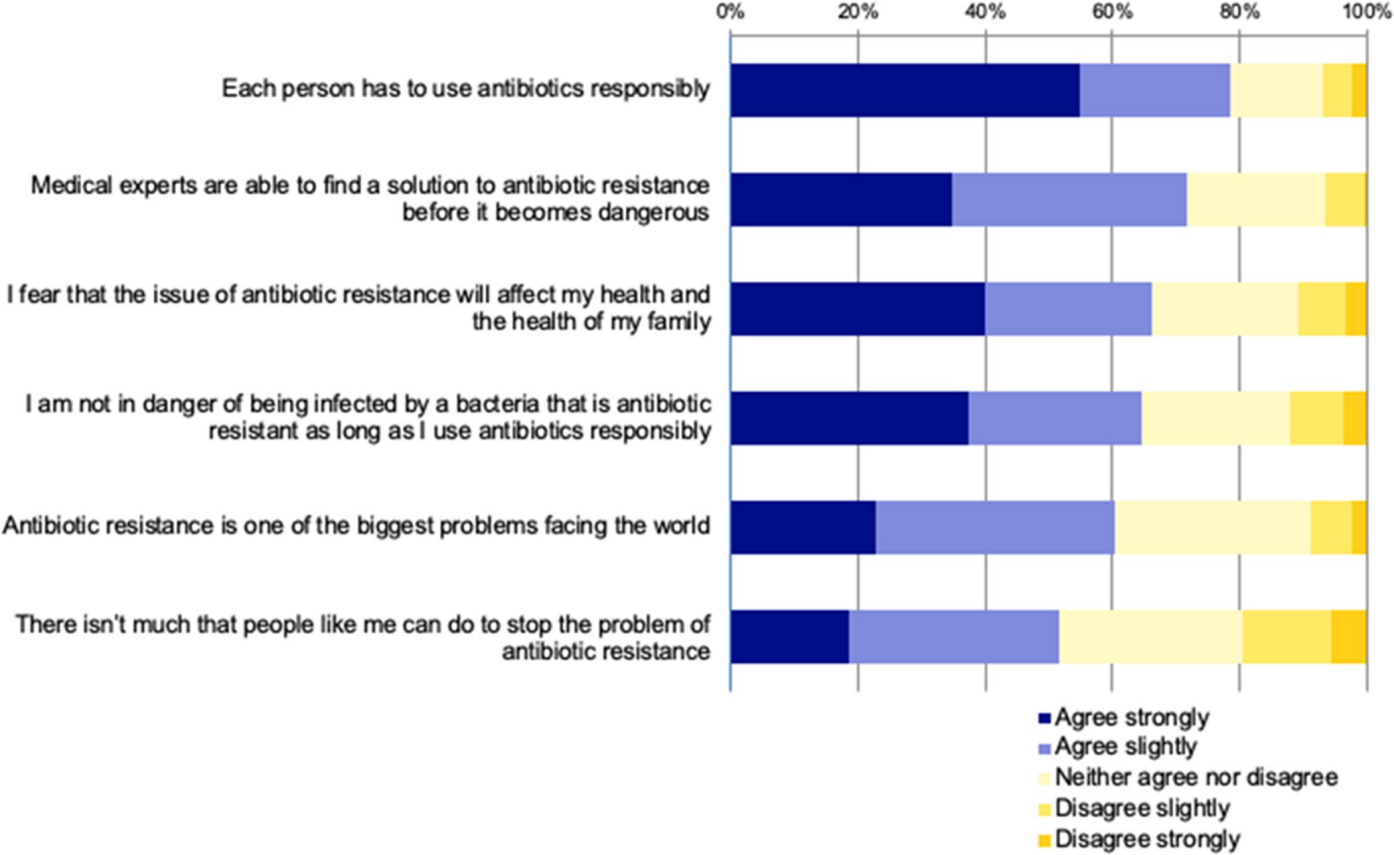
Participants’ attitudes towards the importance of the issue of antibiotic resistance.

Student responses to survey items related to attitudes towards antibiotic resistance and its impacts (Survey Q15) are summarised in Fig 3.

Findings apparent in Fig 3 include general agreement (78.4% of sample) that individuals need to use antibiotics responsibly, but also a level of complacency in that 71% of the sample thought that medical research would solve the resistance problem before it became dangerous. However, most also agreed to some extent that antibiotic resistance could impact their own health or that of their family, while just over half the sample considered that they could not do much to stop the problem of antibiotic resistance.

Congruent results were apparent from the oral questionnaire responses to question 4 (Table 10). When asked about the impact of antibiotic resistance and whether it was a serious problem, about 70% of the sample of students recognised that it was, although less than a third could articulate valid reasons as to why this was the case. A few students made reasonable attempts, suggesting that “Yes, with the development of bacteria is difficult to get rid of …”. Those who responded generally did not elaborate on their responses, but when they did it was generally with a misconception: “No, because it develops a person’s immunity, making it difficult to get sick”.

**Table 10:** Findings pertaining to Question 4: What is the impact of antibiotic resistance and is it a serious problem?.

	Yes		No	
	Reason	Count	Reason	Count
Valid	Because the treatment will become more difficult/ body will not cure	16		
	Because the antibiotics will become ineffective	11		
	Because the strength of the bacteria will increase	7		
	Because we will require a new stronger antibiotic	2		
Invalid	Because the body will no longer accept the antibiotic	5	Because it destroys diseases	8
	Because antibiotics can be harmful and have side effects	4	Because it increases the body’s immunity	6
	Because it reduces the body’s immunity	3	Because antibiotics are beneficial for the body	4
Unclear	Because it has negative side effects on and can harm the body	7	Because the bacteria are familiar to the body	1
	Because the enemy become non-effective	1		
	Because it can cause other diseases	1		
Missing		18		9
Total*				

*Only two respondents answered I don’t know although as shown in previous table, 18 could not define the concept antibiotic resistance.

**Total is 103; One respondent indicated that antibiotic resistance can be a problem in some situations but not in others; another respondent did not answer the question; Two respondents answered “I don’t know”.

One recurring misconception reflected in around a quarter of responses was the framing of resistance as a positive thing (see the Invalid reasons under “No” in Table 10). One student in particular expressed that “antibiotics resistance cannot be a problem; if anything, it is the cure for the body”. It is common in many Arabic countries to associate the idea of “resistance” with noble actions such as defending one’s country. It is interesting, if somewhat speculative, to consider whether there may be any association between these two ideas.

### Perceptions about learning about antibiotic resistance in school

A large majority of students surveyed (70.7%) felt that it was important to learn about antibiotic resistance in school (Survey Q17). Seventy percent of the 952 participants (N = 651) responded to the open item “Why do you think it’s important to learn about the use of antibiotics in schools?” with 29% of respondents mentioning raising awareness about the appropriate use of antibiotics for individuals and in society. So, there appeared, perhaps as a result of completing the survey, that the majority of students were aware that antibiotic misuse was a significant problem in Oman. A much smaller group (8%) responded in terms of disease prevention generally responding simply: “To prevent disease”. Surprisingly, given the number of responses, there appeared to be no misconceptions reported, although a small number of students (5) responded “I don’t know”. Finally, when asked in the oral questionnaire if they would like to know more about antibiotics and antibiotic resistance, the vast majority (86%) responded “Yes” but 18 responded “No”. Of the 18, 3 had stated that they would use antibiotics without a prescription.

### What is learnt at school?

Some exploration of what students learnt at school about antibiotics was undertaken via an analysis of textbooks and a question in the oral questionnaire. Textbook analysis indicated that the only mention of antibiotic resistance appeared in the grade 12 Science and Environment textbook and comprised a short (just over 500 words in English) section titled “Problems with antibiotics” that also addressed side effects and allergies. A brief diagram and text explanation were provided indicating that bacteria can become resistant to antibiotics and multiply, rendering the antibiotic ineffective. This was followed by general advice about how to safely take medical drugs, including a brief sentence indicating that noncompliance with instructions leads to resistant diseases, but with no specific mention of the term antibiotics in that advice section. There were no discussion questions, activities, or references to the severity and importance of the growing concern about antibiotic resistance in this section of the textbook.

Table 11 shows the responses to the oral questionnaire “What did you learn about antibiotics in school?”

**Table 11:** Findings pertaining to Question 6: What did you learn about antibiotics in school?.

	Accurate	Count	Misconceptions	Count
Definitions	Antibiotics are living creatures	3		
Function and Operation of antibiotics or antibiotics resistance	Antibiotics resist bacteria	11	The body produces more antibodies to destroy the disease	1
	Antibiotics are medications that fight disease	4	That antibiotics kill viruses	1
	Bacteria can resist antibiotics	1	Antibiotics are analgesics	2
Behaviour regarding use of antibiotics	Antibiotics should only be used when prescribed by a doctor	17	Antibiotics can only be used once	1
	Antibiotics should not be used frequently	2		
	Antibiotics are only used in emergencies and specific situations	3		
Unspecified	16			

Although there was clearly some attrition by this last question, Table 11 suggests that the majority of those who did respond to this question provided reasonably accurate accounts of antibiotics and antibiotic resistance.

## Discussion and conclusions

Our findings suggest that there are knowledge gaps or misconceptions among a significant proportion of the student sample from Oman that could lead to the inadvertent misuse of antibiotics. This issue is of concern given the high level of antibiotic consumption identified in this study, possibly attributable to the easy availability of broad-spectrum antibiotics and lack of antimicrobial stewardship programs identified by Al-Malky et al [27]. The view that antibiotics could be used to treat colds, flu and symptoms of fever was common among students. Of the four conditions most frequently endorsed by the students as treatable with antibiotics, colds and headaches are not generally caused by bacteria, while some throat infections and fevers may be, but often have other causes such as viruses. Furthermore, when explaining resistance, students tended to focus on the host developing resistance rather than the pathogen. These misconceptions are consistent with reports from other contexts (e.g. [11,13]) including a previous small-scale study in Oman [28]. Of particular interest in this study is the confusion between the concepts of analgesic and antibiotic, which has not, to our knowledge, been previously documented. Given the potential for misuse of antibiotics as analgesics for pain relief, this finding is of concern, and, because it has not previously been identified, there may be a context-specific explanation warranting further exploration. Certainly, it is important that this misconception is addressed.

The findings of most concern, however, were those relating to the misuse of antibiotics including non-completion of courses and obtaining antibiotics from non-medical sources. Again, these have been reported elsewhere, including other territories in the Middle East and Gulf region [16,25].

Overall, the findings indicate a number of worrying gaps in knowledge which may influence behaviours in ways that could result in a failure to mitigate potential antibiotic resistance in Oman. However, they also offered a sense that individuals might treat antibiotics with care if these gaps were addressed.

Clearly these findings indicate the need to educate the general public of Oman about the modes of action and use of antibiotics. Educational campaigns such as the GRACE (Genomics to combat Resistance to Antibiotics for Community acquired lower respiratory tract infection) open-access e-Learning curriculum have been developed around the world and are showing promise (see [34,35]. International colloquia (e.g. [36]) have developed recommended strategies for educating the public about antibiotic resistance. However, a rigorous systematic review of interventions aiming to change awareness and behaviours relating to antimicrobials concluded that although evidence for the impact of educational interventions on the general public was equivocal, “interventions targeting schoolchildren and parents have notable potential” [37 p. 1464].

It appears, therefore, that educational responses to the widespread misconceptions about antibiotics detected in this study need to focus on school students. A review of initiatives to improve antibiotic use conducted with school-aged children [38] indicated that teachers find this topic difficult to teach. The authors of the review suggested that effective mechanisms for enhancing learning in this topic included peer teaching and use of the e-Bug program developed by Public Health England [39], a resource-rich online educational program involving a consortium of 26 international partners aimed at primary and secondary teachers and students. The e-Bug resources are available in 25 languages and are available in Turkey and Saudi Arabia [38] but we are not aware of the use of this resource in Oman. If it has not been adopted, there may be value in Oman joining the consortium or adopting/adapting relevant resources. If this program is currently being used, evaluation of its use, efficacy and transferability could further enhance Oman’s response to antibiotic resistance.

At present, although the Omani school curriculum does contain some information about antibiotics, the findings from this study suggest the information may be either insufficient or that many students miss out on receiving it due to subject choices. Therefore, there could be an argument for introducing this topic in earlier grades when science is compulsory.

The relevant textbook section that we examined was transmissive, brief and all the advice about taking medications phrased in terms of medical drugs in general. Hence, the timing, depth and teaching methods related to antibiotic use in the secondary school curriculum and associated texts in Oman seems a potentially valuable point of response to the issue of antibiotic resistance in the country. Given the importance of this issue, it seems appropriate for the Ministry of Education to review the placement and content of the information on antibiotics in the curriculum, particularly as over 70% of students viewed this topic as important and ranked the school curriculum quite highly as their present source of information about antibiotics. As a point of comparison, in a similar study of antibiotic use among secondary students in Portugal [34], the researchers suggested that teaching of infectious diseases emphasising bacterial and viral pathogens and antibiotic use should occur in all curricular areas in the 9^th^ and 12^th^ grades, that education about use of medications should take place much earlier and that relevant programs and resources should be designed and tested in schools.

Some results of specific teaching interventions for students the same age as those in this study have been published, though we are aware of none from the Gulf Countries. A specific short teaching intervention of a slide show followed by a discussion about appropriate antibiotic use in Year 9 students in Portugal found significant increases in knowledge [34], however no delay post-test was conducted. A more substantial and immersive intervention of taking 42 high school students for a week-long laboratory-based experience at a university in Portugal [40] enhanced students’ knowledge and awareness of antibiotic use and resistance and may also enhance students’ wider scientific literacy and skills as well as their curiosity about the topic. However, it is difficult to see how this might be scaled up to the whole school cohort.

In a similar vein, interactive ‘‘hands–on’’ workshops targeting the issue of antimicrobial resistance conducted by staff from the University of Turin with over 1,200 primary school children aged 9 to 11 years old in their schools resulted in significant gains in understanding [41], indicating the value of relevant learning in younger years. The inclusion of laboratory-based activities, such as simple bioassays, offers students a different experience of a topic that is often delivered didactically; and if university visits or linkages are not viable or appropriate, modifications of such activities may be possible in Omani schools, which generally have good laboratory facilities. However, this would require appropriate professional development, particularly in risk assessment for teachers. Each secondary school in Oman is allocated a trained nurse, who may have sufficient medical training to work with teachers to develop pedagogical content knowledge in this area. Thus, drawing on aspects of this model may be feasible.

Antibiotic resistance as a topic is not only a vital socio-scientific issue to address in any education system but it is also relevant to all students as all will have experienced taking antibiotics or seen a close relative doing so. The lack of understanding among Omani adolescents about the nature and use of antibiotics, coupled with the critically important problem of antibiotic resistance and the identified potential of education targeted at school-aged young people suggests that efforts directed at this aspect of the Oman school curriculum should be a high priority.

## Supporting information

S1 Appendix Survey. (DOCX)

S2 Appendix Oral questionnaire protocol. (DOCX)
