# Supplementary material for: Omani senior secondary school students’ knowledge of and attitudes to antibiotic resistance
Source: PLoS One. 2022 Feb 25;17(2):e0264500. doi: 10.1371/journal.pone.0264500 (PMC8880815; doi:10.1371/journal.pone.0264500)
Supplement: S1 Appendix — (DOCX) [file pone.0264500.s001.docx]

# S1 Appendix A: Survey

This questionnaire aims at surveying the current practices and attitudes towards the uses of antibiotics amongst school students between 15-17 years of age in the Sultanate of Oman. By completing this survey, you will provide important information to help each of the ministry of education and the ministry of health in the Sultanate to develop a strategy to face this problem. All information provided will be dealt with confidentiality and will only be used for the purposes of this research. Before completing the survey, please fill in the data sheet below

| Social Gender: Male Female  Age 15 16 17 Other: write it here -----------------  Place where you live: City Outside the city (in a village or an agricultural area)  Educational governorate: ------------ |
| --- |

**Antibiotics** are defined in this survey as the substances that discourage the growth of some microscopic organisms through entering it, thus causing trouble in its metabolism or damaging it.

**The survey**

Place a circle around the best answer for the following questions

1. When was last time you had consumed antibiotics?
   1. During the past month
   2. During the past six months
   3. During the past year
   4. More than a year ago
   5. I never consumed it
   6. I don’t remember/I don’t know (if this is your answer, go straight to question number 6)
2. On that occasion, did you take antibiotics from a doctor/through a medical script?
   1. Yes
   2. No
   3. I don’t remember/I don’t know
3. On that occasion, did you get instructions from a doctor or pharmacist about how to consume these antibiotics?
   1. Yes, I got instructions (for example: with food, for seven days)
   2. No
   3. I don’t remember/I don’t know
4. On that occasion, did you get instructions from your parents about how to consume these antibiotics?
   1. Yes, I got instructions (for example: with food, for seven days)
   2. No
   3. I don’t remember/I don’t know
5. On that occasion, where did you/your parents obtain these antibiotics from?
   1. The pharmacy
   2. Internet
   3. Friends/relatives
   4. I had them from a previous time
   5. Somewhere else (specify …………………………)
   6. I don’t remember
6. When do you think you should stop having antibiotics after the start of the treatment?
   1. When I feel better
   2. When I have antibiotics according to instructions
   3. I don’t know
7. Do you believe this expression is correct or wrong: “It is OK to use antibiotics that were prescribed to someone else as long as the disease that is being treated for is the same.”
   1. Correct
   2. Wrong
   3. I don’t know
8. Which of the following diseases can be cured with antibiotics? Place a circle around all the answers that you think are correct
   1. Acquired Immune Deficiency Syndrome (AIDS)
   2. Gonorrhoea
   3. Urinary tract infection
   4. Diarrhoea
   5. Cold
   6. Fever
   7. Malaria
   8. Measles
   9. Skin infection/wound infection
   10. Throat infection
   11. Body aches
   12. Headache
   13. Tuberculosis
   14. Brucellosis/Intestinal worms
9. Have you ever heard of the term “Antibiotic resistance”? a. Yes b. No

If you answered yes, from where did you hear about this term? Circle all possible answers

1. Doctor
2. Nurse
3. Pharmacist
4. School curriculum
5. Friend or relative (including social media)
6. Media (TV, radio, newspapers or other)
7. Awareness campaign
8. Other
9. I don’t remember
10. Have you ever heard about the term “Super Bug”? a. Yes b. No

If you answered yes, from where did you hear about this term? Circle all possible answers

1. Doctor
2. Nurse
3. Pharmacist
4. School curriculum
5. Friend or relative
6. Media (TV, radio, newspapers or other, in addition to social media)
7. Awareness campaign
8. Other
9. I don’t remember
10. Have you ever heard about the term “drug resistance”? a. Yes b. No

If you answered yes, from where did you hear about this term? Circle all possible answers

1. Doctor
2. Nurse
3. Pharmacist
4. School curriculum
5. Friend or relative
6. Media (TV, radio, newspapers or other, in addition to social media)
7. Awareness campaign
8. Other
9. I don’t remember
10. Have you ever heard about the term “antimicrobial resistance”? a. Yes b. No

If you answered yes, from where did you hear about this term? Circle all possible answers

1. Doctor
2. Nurse
3. Pharmacist
4. School curriculum
5. Friend or relative (including social media)
6. Media (TV, radio, newspapers or other)
7. Awareness campaign
8. Other
9. I don’t remember
10. Place a circle around “correct” or “wrong” to indicate your answer to the following expressions

| 1. Antibiotic resistance occurs when your body becomes resistant to the positive effects of antibiotics, and antibiotics no longer work as they should | Correct | Wrong |
| --- | --- | --- |
| 1. A lot of types of infections have become increasingly resistant to antibiotics | Correct | Wrong |
| 1. If a bacterium is resistant to antibiotics, then the cure for the infection caused by the bacterium becomes extremely difficult or impossible | Correct | Wrong |
| 1. Antibiotics resistance is an issue that could affect me or my family | Correct | Wrong |
| 1. Antibiotics resistance is an issue in other countries but not in Oman | Correct | Wrong |
| 1. Antibiotics resistance is an issue for those who consume antibiotics regularly | Correct | Wrong |
| 1. Bacteria that are antibiotic resistant can be passed from one person to another | Correct | Wrong |
| 1. Infections caused by bacteria that are antibiotics resistant can make some medical procedures such as surgery, organs transplant, and cancer treatments much more dangerous than they were | Correct | Wrong |
| 1. Antibiotic resistance can be overtaken by increasing the dose of the medication | Correct | Wrong |
| 1. It is possible to cure diseases related to viruses through using antibiotics | Correct | Wrong |

1. To what extent do you agree with the following statements related to how to address the issue of antibiotic resistance? Circle one answer for each of the provided statements:

| 1. People have to use antibiotics when they are prescribed by a doctor only | Agree strongly | Agree slightly | Neither  agree nor disagree | Disagree slightly | Disagree strongly |
| --- | --- | --- | --- | --- | --- |
| 1. Farmers need to give lesser amounts of antibiotics to food producing animals | Agree strongly | Agree slightly | Neither  agree nor disagree | Disagree slightly | Disagree strongly |
| 1. People must not keep leftover antibiotics and must not use it on other occasions to treat other illnesses | Agree strongly | Agree slightly | Neither  agree nor disagree | Disagree slightly | Disagree strongly |
| 1. Parents must ensure that their kids are immunised on time | Agree strongly | Agree slightly | Neither  agree nor disagree | Disagree slightly | Disagree strongly |
| 1. People must wash their hands constantly | Agree strongly | Agree slightly | Neither  agree nor disagree | Disagree slightly | Disagree strongly |
| 1. Doctors must prescribe antibiotics only when necessary | Agree strongly | Agree slightly | Neither agree nor disagree | Disagree slightly | Disagree strongly |
| 1. The government must provide rewards for those who develop new antibiotics | Agree strongly | Agree slightly | Neither  agree nor disagree | Disagree slightly | Disagree strongly |
| 1. Pharmaceutical companies must develop new antibiotics | Agree strongly | Agree slightly | Neither  agree nor disagree | Disagree slightly | Disagree strongly |

1. To what extent do you agree with the following statements? Circle one answer for each of the provided statements?

| 1. Antibiotic resistance is one of the biggest problems facing the world | Agree strongly | Agree slightly | Neither  agree nor disagree | Disagree slightly | Disagree strongly |
| --- | --- | --- | --- | --- | --- |
| 1. Medical experts are able to find a solution to antibiotic resistance before it becomes dangerous | Agree strongly | Agree slightly | Neither  agree nor disagree | Disagree slightly | Disagree strongly |
| 1. Each person has to use antibiotics responsibly | Agree strongly | Agree slightly | Neither  agree nor disagree | Disagree slightly | Disagree strongly |
| 1. There isn’t much that people like me can do to stop the problem of antibiotic resistance | Agree strongly | Agree slightly | Neither  agree nor disagree | Disagree slightly | Disagree strongly |
| 1. I fear that the issue of antibiotic resistance will affect my health and the health of my family | Agree strongly | Agree slightly | Neither  agree nor disagree | Disagree slightly | Disagree strongly |
| 1. I am not in danger of being infected by a bacteria that is antibiotic resistant as long as I use antibiotics responsibly | Agree strongly | Agree slightly | Neither  agree nor disagree | Disagree slightly | Disagree strongly |

1. Do you think that antibiotics are used in a large scale in the agricultural sector (including the sector of food-producing animals) in Oman?
   1. Yes
   2. No
   3. I don’t know
2. Do you think it is important to learn about the use of antibiotics in schools?
   1. Yes
   2. No
   3. I don’t know

Why do you think it’s important to learn about the use of antibiotics in schools?

……………………………………………………………………………………………….

……………………………………………………………………………………………….

……………………………………………………………………………………………….

……………………………………………………………………………………………….

Thank you very much for your cooperation in responding to this survey
