# Supplementary material for: Omani senior secondary school students’ knowledge of and attitudes to antibiotic resistance
Source: PLoS One. 2022 Feb 25;17(2):e0264500. doi: 10.1371/journal.pone.0264500 (PMC8880815; doi:10.1371/journal.pone.0264500)
Supplement: S2 Appendix — (DOCX) [file pone.0264500.s002.docx]

# S2 Appendix B: Oral Questionnaire

1. Can you tell me what you know about antibiotics - what they are and how they work?’ (research question 2)
2. ‘Would you take antibiotics without a prescription? Why/why not? (research question 1)
3. Do you understand what is meant by antibiotic resistance? How does this develop?’ (research question 2)
4. What is the impact of antibiotic resistance and is it a serious problem? (research question 3)
5. Would you like to know more about antibiotics and antibiotic resistance? What did you learn about antibiotics in school?
